# Case Report: Appendiceal neurofibroma associated with neurofibromatosis type 1: a rare case and systematic review of the literature

**DOI:** 10.3389/fsurg.2026.1781055

**Published:** 2026-03-27

**Authors:** Yan-Feng Jiang, Xin-Zhang Li, Ping-An Wang, Xin-Jun Li

**Affiliations:** 1 The First General Surgery Ward, East Campus of Binzhou People’s Hospital, Binzhou, China; 2 Department of Pathology, Binzhou People’s Hospital, Binzhou, China

**Keywords:** appendiceal neurofibroma, neurofibromatosis type 1, case report, literature review, appendix

## Abstract

**Background:**

Appendiceal neurofibroma is exceedingly rare, with fewer than 20 cases reported globally to date. It is most commonly observed in patients with neurofibromatosis type 1 (NF1), and both clinical and imaging characteristics lack specificity.

**Case summary:**

A 31-year-old male presented with a 10-day history of fever; a right-lower-quadrant mass was detected during outpatient evaluation, prompting hospital admission. Abdominal CT revealed a 6.9 × 4.2 cm mass in the appendiceal region, and ultrasound showed the lesion was connected to the appendiceal lumen. The patient underwent appendectomy, partial cystectomy, and partial omentectomy. Histopathological examination revealed spindle cell bundles, with positive S-100 and SOX-10 staining, and Ki-67 expression <2%. The final diagnosis was appendiceal neurofibroma with focal atypical hyperplasia.

**Literature search method:**

A systematic search of PubMed and Embase was conducted to identify previously reported cases of appendiceal neurofibroma.

**Results:**

A total of 16 cases of appendiceal neurofibroma were identified from the English-language literature through PubMed and Embase (excluding the present case). Among the 16 cases reporting gender, 11 were male and 5 were female. The median age of the 16 cases with reported ages was 51 years (range, 19–74 years). Of the 16 cases, 14 were associated with NF1, and 2 were isolated cases of appendiceal neurofibroma. At least 12 cases (75%) presented with right lower quadrant pain or acute appendicitis-like symptoms, while the remaining cases were characterized by abdominal masses, gastrointestinal bleeding, or incidental findings on imaging.

**Conclusion:**

This case of appendiceal neurofibroma presented primarily with persistent fever and lacked typical abdominal pain. Imaging revealed an appendiceal mass closely associated with the bladder. After appendectomy combined with partial cystectomy, the diagnosis of neurofibroma was confirmed by pathology. The patient recovered well postoperatively, and at 6-month telephone follow-up no symptoms suggestive of recurrence were reported; however, no interval imaging was performed.

## Introduction

Neurofibromatosis type 1 (NF1) is a genetic tumor predisposition syndrome, and gastrointestinal involvement has been reported in a subset of patients (1). However, appendiceal neurofibroma is extremely rare and has only been described in sporadic case reports. It is often misdiagnosed as acute appendicitis or other appendiceal tumors due to its atypical clinical and imaging features (2). Here, we report a case of a patient who was admitted with persistent fever as the main complaint, after a right lower-quadrant mass was detected during outpatient evaluation. Abdominal CT revealed an appendiceal mass closely abutting the bladder. The patient was successfully treated with open appendectomy combined with partial cystectomy and partial omentectomy, and pathology confirmed the diagnosis of neurofibroma. The postoperative recovery was smooth. Additionally, we reviewed 16 previously reported cases of appendiceal neurofibroma in the English literature, summarizing their clinical, imaging, and surgical characteristics to better define the spectrum of this rare disease and provide practical guidance for diagnosis and management.

## Case presentation

**Figure 1:**
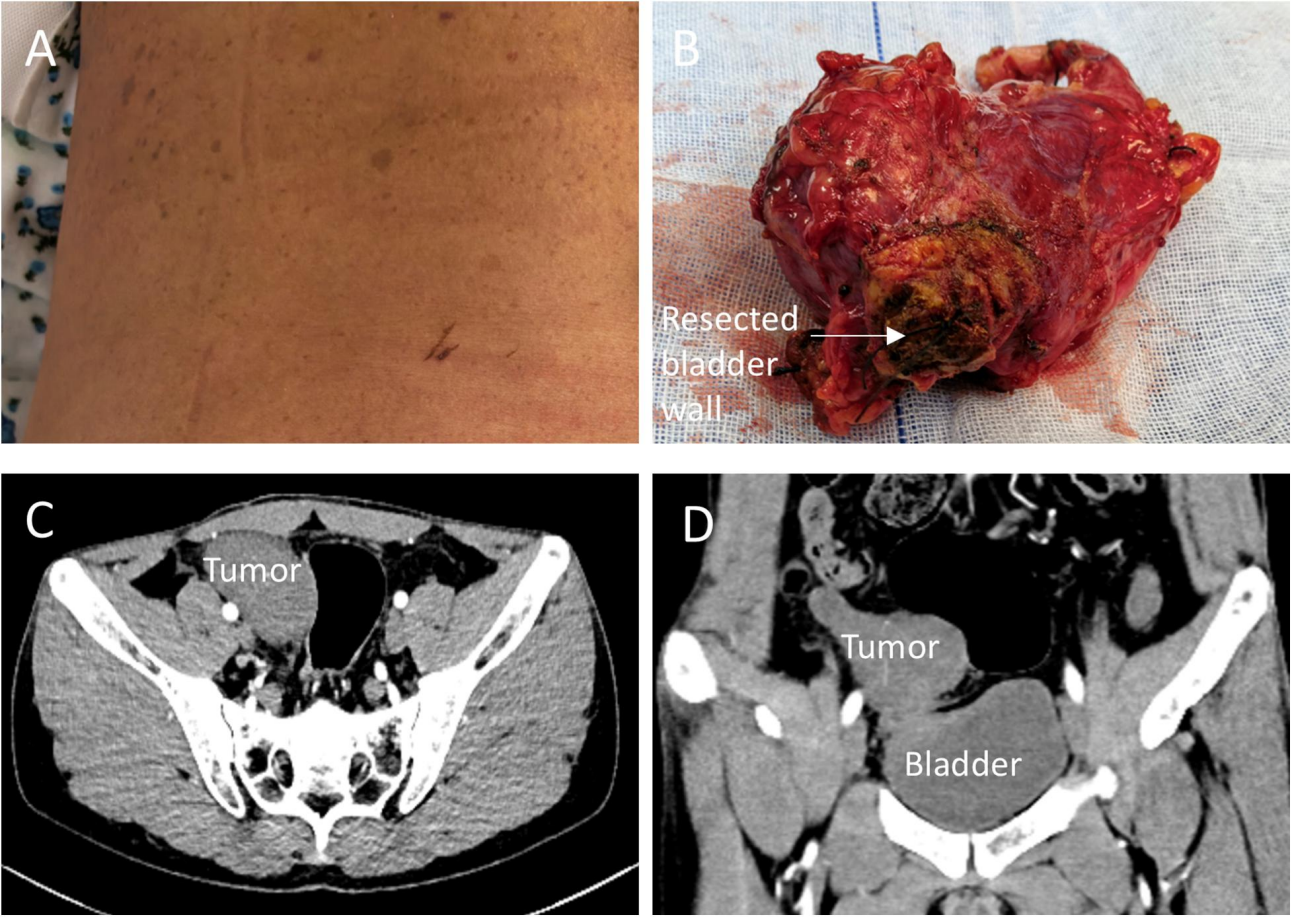
Clinical, imaging, and gross features of appendiceal neurofibroma in an NF1 patient. **(A)** Café-au-lait macules. **(B)** Gross specimen showing an appendiceal tumor with an adherent segment of bladder wall (resected en bloc due to dense adhesion). **(C,D)** Contrast-enhanced CT demonstrating an appendiceal mass abutting/involving the urinary bladder.

A 31-year-old man with NF1 and no family history was admitted for a 10-day history of fever. A palpable right lower-quadrant mass was identified during outpatient evaluation one day prior to admission, prompting hospitalization. Café-au-lait macules had been present since childhood (Figure 1). On admission, his temperature was 38.5°C, heart rate 98 beats/min, and blood pressure 118/76 mmHg. The abdomen was soft, with mild tenderness in the right lower quadrant and no rebound tenderness. A poorly demarcated mass measuring approximately 7 × 5 cm was palpated inferolateral to McBurney's point. Cutaneous examination revealed multiple café-au-lait macules (>15 mm) and subcutaneous nodules, meeting clinical criteria for NF1.

Laboratory testing showed a white blood cell count of 6.08 × 10⁹/L with 70% neutrophils and a C-reactive protein level of 22.94 mg/L. Serum tumor markers (CEA, CA19-9, and CA125) were within normal limits. Contrast-enhanced abdominal CT demonstrated a well-circumscribed mass in the appendiceal region measuring 6.9 × 4.2 cm. The lesion was relatively homogeneous and closely abutted the anterior bladder wall; no enlarged mesenteric lymph nodes were identified (Figure 1). Ultrasonography revealed a mixed-echoic mass with fine internal septations and a tract communicating with the appendiceal lumen, with scant intralesional blood flow. Colonoscopy identified a small rectal polyp, which was diagnosed as inflammatory hyperplasia; no abnormality was observed in the cecum.

After preoperative assessment, exploratory laparotomy was performed. An oval mass (approximately 7 × 6 × 4 cm) arising from the appendix and mesoappendix was found, with dense adhesions to the bladder dome and a portion of the greater omentum. The appendiceal base and cecal wall appeared intact. The adherent portion of the greater omentum was partially resected (partial omentectomy), and appendectomy was performed 0.5 cm from the appendiceal base. The involved segment of the anterior bladder wall was resected *en bloc* and repaired with a two-layer closure. The operative time was 120 min, and estimated blood loss was 100 mL; no transfusion was required, and no intra-abdominal drain was placed.

**Figure 2:**
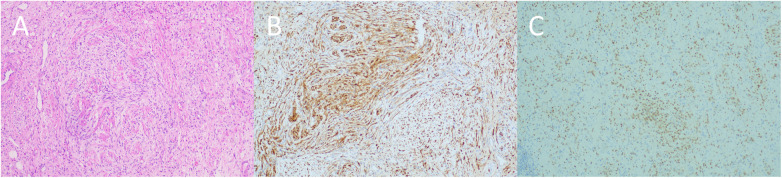
Histopathological features of the appendiceal neurofibroma. **(A)** Hematoxylin and eosin (HE) staining showing characteristic spindle cell arrangement of neurofibroma. **(B)** S100 immunohistochemical staining demonstrating strong positivity in tumor cells, supporting the diagnosis of neurofibroma. **(C)** SOX10 immunohistochemical staining showing neurogenic features, further confirming the diagnosis (all at 100× magnification).

Gross examination showed a fusiformly thickened appendix with a smooth serosal surface and a gray-white, solid cut surface. Histopathology revealed interlacing bundles of spindle cells with wavy nuclei in a myxoid stroma, without conspicuous mitotic activity. Immunohistochemistry showed diffuse positivity for S-100 and SOX10, positivity for CD56, and equivocal focal staining for synaptophysin; CKpan and EMA were negative. The Ki-67 labeling index was <2% (Figure 2). CD34 and c-Kit (CD117) immunostaining was not performed in this case. Taken together, the findings supported a diagnosis of appendiceal neurofibroma with focal atypical hyperplasia.

**Figure 3:**
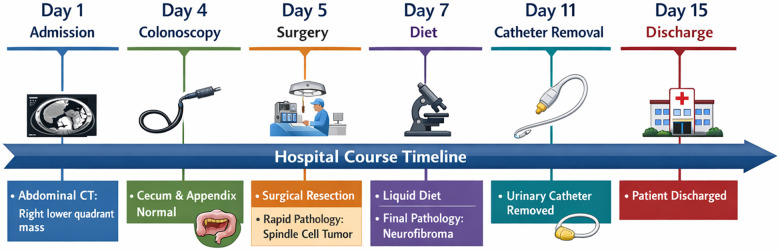
Timeline of the patient's hospital course, from admission (Day 1) to discharge (Day 15), highlighting key clinical events, surgical treatment, pathology results, and recovery milestones. This timeline includes abdominal CT, colonoscopy, surgery, pathological analysis, dietary adjustments, and catheter removal, providing a comprehensive overview of the patient's treatment and recovery process.

The patient ambulated on postoperative day 1, resumed a semi-liquid diet on day 3, and was discharged on May 8, 2025 (postoperative day 9) without fever and with normal urination (Figure 3). Telephone follow-up at 6 months after discharge indicated that the patient remained asymptomatic, with no fever, abdominal pain, urinary symptoms, or other complaints suggestive of recurrence. No interval imaging or laboratory reassessment was performed during follow-up.

Patient Perspective: The patient reported marked relief of discomfort after surgery and expressed reassurance after being informed of the benign nature of the lesion. During telephone follow-up, the patient indicated good recovery and understanding of the follow-up plan.

## Literature search and study selection

**Figure 4:**
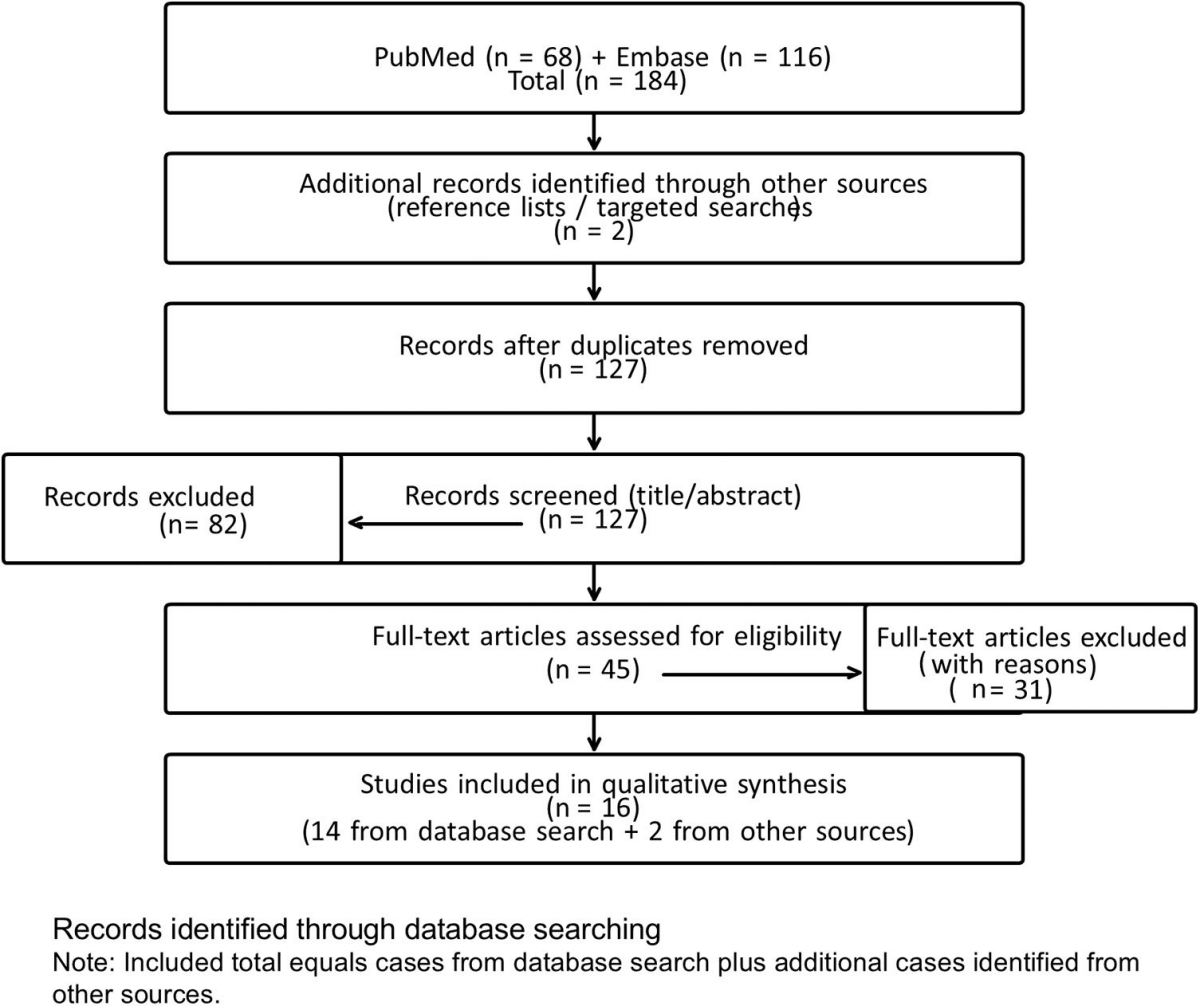
PRISMA-like flow diagram of the literature search for appendiceal neurofibroma (ANF). Records identified from PubMed (n = 68) and Embase (n = 116), with 2 additional records identified through other sources, giving 184 total records. After duplicate removal, 127 records were screened; 82 were excluded, 45 full-text articles were assessed for eligibility, and 31 were excluded, leaving 16 studies for qualitative synthesis.

A systematic literature search was conducted to identify previously reported cases of appendiceal neurofibroma (Figure 4). We searched PubMed and Embase for articles published between 1975 and December 2025 and restricted the search to studies published in English. For both databases, we combined controlled vocabulary (MeSH/Emtree) and free-text terms related to the appendix (e.g., “appendix”, “appendiceal”) and neurogenic tumors (e.g., “neurofibroma”, “neurogenic tumor”, “nerve sheath”), together with filters for case reports and small case series. In addition, high-sensitivity supplementary queries were used to capture inconsistently indexed reports and NF1-related cases. The complete search strategies for each database are provided in Supplementary Table S1.

The initial search identified 184 records (68 from PubMed and 116 from Embase), which were screened by title/abstract and full text according to predefined eligibility criteria. Studies were included if they met all of the following criteria: (1) histopathological confirmation of a neurofibroma or neurofibromatous lesion arising in the appendix; (2) single case reports or small case series in which individual appendiceal cases could be clearly identified; and (3) provision of sufficient clinical, imaging, surgical and/or follow-up information to allow meaningful comparison. We excluded non-English publications without extractable clinical details and reports with overlapping cases. Manual screening of reference lists and targeted supplementary searches identified two additional relevant reports that were not indexed in the original PubMed/Embase query. In total, 16 individual cases of appendiceal neurofibroma from the English-language literature were included in the qualitative synthesis and are summarized together (Table 1).

**Table 1:** Reported cases of appendiceal neurofibroma in the English-language literature (excluding the present case).

First author (year)	Age/Sex	NF1	Presentation	Imaging/Endoscopy	Operation	Outcome/Follow-up
Rosenberg (3)	33/F	Yes	Incidental finding during cesarean section	US (preop): mass not visualized; incidental appendiceal mass identified intraoperatively	Appendectomy	Uneventful immediate recovery; long-term follow-up NR
Sugimoto (4)	67/M	Yes	Abdominal pain	CT: abdominal mass	Ileocecal resection with D2 regional lymph node dissection	Recovered
Olsen (5)	24/M	No	Abdominal pain	NR	Appendectomy	Recovered; no recurrence reported (follow-up NR)
Merck (6)	24/M	Yes	Abdominal pain	NR	Appendectomy	NR
Ozaki (7)	51/M	Yes	Abdominal pain	CECT: an inflamed, diffusely thickened appendix with a periappendiceal abdominal abscess	Periappendiceal abscess drainage; appendectomy	NR
Van de Steen (8)	74/M	Yes	Abdominal pain	CECT: appendiceal wall thickening	Appendectomy	Recovered
Sakashita (9)	68/M	Yes	Asymptomatic	CT: abdominal mass	Laparoscopic ileocecal resection with lymph node dissection	Recovered
Greenberg (10)	52/F	Yes	Abdominal pain	CT: appendiceal thickening	Appendectomy; partial cystectomy	Recovered
Guo (11)	62/F	Yes	Abdominal pain; Mass	CECT: mass	Right hemicolectomy	Recovered
Jeong (12)	61/M	Yes	Abdominal mass	CT: thickened appendiceal wall; colonoscopy: swollen appendiceal orifice	Appendectomy; partial cecal resection	Recovered
Samuel (13)	19/M	Yes	Abdominal pain; Mass	MRI: appendiceal mass	Appendectomy	NR
Wilson (14)	24/M	Yes	Abdominal pain; Nausea; Vomiting	CT: appendiceal mass	Ileocecectomy	Recovered
Komo (15)	62/M	Yes	Abdominal pain	CECT: an enlarged and diffusely thickened appendix	Laparoscopic cecectomy	Recovered
Farag (16)	51/F	Yes	Nausea; Vomiting; Abdominal pain	CT: appendiceal thickening	Ileocecectomy	Recovered
Ohnishi (17)	59/M	No	Bloody stools	Colonoscopy: tumor at appendiceal orifice	Endoscopic biopsy	No recurrence at 10 mo
Alkhatatneh (18)	51/F	Yes	Abdominal pain; Nausea; Vomiting	CT: appendiceal mucocele	Ileocecal resection	Recovered

NF1, neurofibromatosis type 1; CT, computed tomography; CECT, contrast-enhanced CT; US, ultrasound; MRI, magnetic resonance imaging; NR, not reported.

## Discussion

Appendiceal neurofibroma (ANF) is extremely rare (11, 13, 19–21). In our pooled analysis of 16 previously reported English-language cases, 14 (87.5%) occurred in patients with NF1 (Table 1). The median age of presentation is approximately 51 years (Table 1), although our case involved a 31-year-old male, highlighting the atypical age range for NF1-related tumors. While most patients present with abdominal pain and symptoms resembling acute appendicitis (10, 13, 20), in our pooled analysis, at least 12 of 16 cases (≈75%) presented with right lower-quadrant pain or an acute appendicitis–like syndrome (Table 1). The present case differed, with persistent fever as the main complaint; a palpable right lower-quadrant mass was detected during outpatient evaluation and confirmed on imaging. This underscores the diagnostic challenge of ANF, as it is often misdiagnosed as appendicitis or other tumors.

Imaging studies typically show an enlarged or thickened appendix (8, 11, 13, 21), often misdiagnosed as appendicitis, diverticulitis, or a mucocele (22). In our patient, CT and ultrasound revealed an appendiceal mass closely adherent to the bladder wall, making preoperative diagnosis particularly challenging. This highlights the importance of considering rare neurogenic tumors when encountering atypical appendiceal masses in NF1 patients and suggests that multiphasic CT or MRI, with a focus on nerve plexus continuity, may aid in preoperative recognition.

Histologically, neurofibromas are classified as localized (solitary), diffuse, or plexiform types. Although most sporadically occurring and localized neurofibromas have an extremely low risk of malignant transformation, neurofibromas in the context of NF1—particularly plexiform neurofibromas—carry an increased risk of malignant transformation and may give rise to malignant peripheral nerve sheath tumors (MPNSTs) (23). In our pooled analysis of 16 published appendiceal cases, 7 (44%) were reported as plexiform neurofibromas, and some lesions showed diffuse involvement of the appendiceal wall (Table 1) (15). In our case, the tumor had well-defined margins and a low Ki-67 index (<2%), suggesting a benign course. However, the presence of atypical hyperplasia raises the concern of a “borderline” state, warranting careful follow-up. Surgical treatment typically involves appendectomy, although more extensive resections, such as right hemicolectomy or ileocecal resection, may be required in some cases (4, 9, 11, 14, 16, 18). In our case, the patient underwent appendectomy combined with partial cystectomy, and had an uneventful recovery, which is consistent with successful outcomes in similar cases. CD34 and c-Kit (CD117) immunostaining were not performed in this case; however, the diffuse S-100 and SOX10 positivity together with the histomorphology strongly supported a diagnosis of neurofibroma.

This review of 16 reported cases, including our own, provides valuable insights into the clinical and surgical management of ANF, despite its limitations, such as the small number of cases and the lack of genetic sequencing in many studies. Nevertheless, this is the first systematic review to collate all reported ANF cases over the past 50 years, offering a structured comparison of diagnostic challenges and surgical approaches. Our findings emphasize the importance of recognizing this rare disease in NF1 patients, especially when clinical symptoms deviate from typical appendicitis presentations.

## Data Availability

The original contributions presented in the study are included in the article/supplementary materials. Further inquiries can be directed to the corresponding author.
