# Supplementary material for: Case Report: Appendiceal neurofibroma associated with neurofibromatosis type 1: a rare case and systematic review of the literature
Source: Front Surg. 2026 Mar 27;13:1781055. doi: 10.3389/fsurg.2026.1781055 (PMC13066255; doi:10.3389/fsurg.2026.1781055)
Supplement: Supplementary file 1 [file Table1.docx]

**Supplementary Table S1: Literature Search Strategies for PubMed and Embase**

| **Database** | **Search Terms** | **Filters** | **High-Sensitivity Supplementary Queries** |
| --- | --- | --- | --- |
| **PubMed** | ("Appendix"[Mesh] OR appendix[Title/Abstract] OR appendiceal[Title/Abstract] OR vermiform appendix[Title/Abstract]) AND ("Neurofibroma"[Mesh] OR neurofibroma[Title/Abstract] OR neurofibromas[Title/Abstract] OR neurogenic tumor[Title/Abstract]) | Case reports[Publication Type] OR case report[Title] OR case series[Title/Abstract] | (appendi*[Title/Abstract]) AND (neurofibrom*[Title/Abstract] OR "neurogenic tumor"[Title/Abstract] OR "nerve sheath"[Title/Abstract]) |
| **Embase** | ('appendix'/exp OR appendix:ti,ab OR appendiceal:ti,ab OR 'vermiform appendix':ti,ab) AND ('neurofibroma'/exp OR neurofibroma:ti,ab OR neurofibromas:ti,ab OR 'neurogenic tumor':ti,ab) | 'case report'/exp OR 'case report':ti OR 'case series':ti,ab | appendi*:ti,ab AND neurofibrom*:ti,ab OR 'nerve sheath':ti,ab OR 'neurogenic tumor':ti,ab |
| **Supplementary Search** |  |  | (appendi*:ti,ab) AND ('neurofibromatosis type 1':ti,ab OR NF1:ti,ab) AND ('case report'/exp OR 'case report':ti) |

窗体顶端

窗体底端
